# Fatigue Life Anisotropy of API 5L X42 Pipeline Steel in Axial Force-Controlled Tests

**DOI:** 10.3390/ma18112484

**Published:** 2025-05-26

**Authors:** Manuel A. Beltrán-Zúñiga, Jorge L. González-Velázquez, Diego I. Rivas-López, Héctor J. Dorantes-Rosales, Carlos Ferreira-Palma, Felipe Hernández-Santiago, Fernando Larios-Flores

**Affiliations:** 1 DIMM, ESIQIE, Instituto Politécnico Nacional, México City 07738, Mexico; mbeltranz@ipn.mx (M.A.B.-Z.); jlgonzalezv@ipn.mx (J.L.G.-V.); hdorantes@ipn.mx (H.J.D.-R.); flariosf1500@alumno.ipn.mx (F.L.-F.); 2 Facultad de Ciencias Químicas Región Veracruz, Universidad Veracruzana, Boca del Río 94294, Mexico; carferreira@uv.mx; 3 ESIME-UA, Instituto Politécnico Nacional, México City 02550, Mexico; fhernandezs@ipn.mx

**Keywords:** fatigue lifetime, anisotropy, pipeline steel, mechanical properties

## Abstract

Fatigue endurance anisotropic behavior was evaluated for an API 5L X42 pipeline steel through axial force-controlled fatigue tests amongst Longitudinal, Diagonal, and Circumferential directions. This study shows that fatigue life anisotropy is mainly controlled by pearlite banding degree (*A_i_*) and ferritic grain orientation (*Ω*_12_). Also, it is foreseen that the observed behavior can be related to the dislocation arrays generated by the cyclic loading in relation to microstructure orientation, and the interactions of the fatigue crack tip with the microstructure during the crack propagation stage.

## 1. Introduction

Marine pipelines and offshore raisers are exposed to fatigue due to cyclic loading induced by ocean currents, slug flow, and other varying loads. One important concern is that these loads are also variable in direction, which induces stress components in different directions in the pipe. Pipeline design codes assume that the mechanical behavior of pipeline steel is isotropic [1,2,3,4,5,6], but experimental evidence indicates that it is not [7,8,9]; therefore, the anisotropic behavior must be considered in the design and structural integrity assessment.

**Figure 1:**
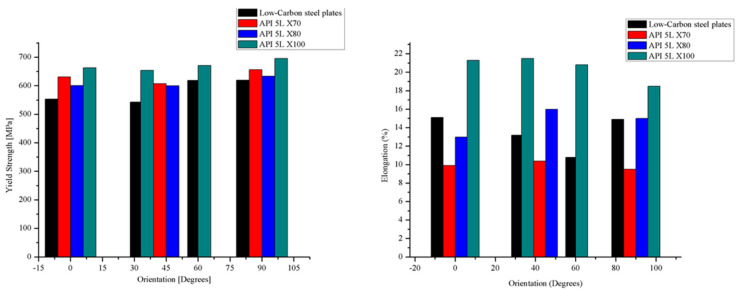
Tensile properties of low-carbon steel plates and pipelines, literature review.

Anisotropy of the fundamental tensile properties of low-carbon steel plates and steel pipelines has been well documented [10,11]. Since the longitudinal direction typically corresponds to the rolling direction, there is a general consensus that the anisotropic behavior in pipeline steels is related to the directionality of the microstructural features introduced by the hot rolling processes stage during the fabrication of the pipe, such as grain elongation, segregation of nonmetallic inclusions, pearlite banding, and crystallographic texture [12,13,14,15,16,17]. Figure 1 shows the tensile behavior reported in the literature for low-carbon steel plates and pipelines, showing the yield strength and elongation as a function of the orientation [9,12,18].

Furthermore, the anisotropic behavior of steel plates and pipes has also been observed in the fracture properties, such as Charpy impact and plane strain fracture toughness, but due to the complex relation between the fracture plane orientation and the orientation of the microstructural features, the results are widespread [18,19,20,21,22,23,24,25,26,27,28,29].

Heiser and Hertzberg [30] evaluated the fatigue crack growth rates of hot rolled steel plates with banded ferrite/pearlite microstructures in L-T, T-S, and S-L orientations, finding that the fatigue crack growth rate is sensitive to the orientation of the microconstituents with respect to the fracture plane. When brittle particles are oriented normally to the applied stress axis, local fracture events would be expected to occur more readily than when they are parallel to the loading direction. These local fractures should lead to faster fatigue crack propagation as well as lower toughness in the short transverse specimens; observations of both higher growth rate and lower toughness were seen in the short transverse specimens. Ohji et al. [31] evaluated the fatigue crack growth behavior of anisotropic rolled steel plate (SB56M) with annealing and spheroidizing heat treatments. They observed that the anisotropy of fatigue crack growth is caused by the banded pearlite microstructure, while nonmetallic inclusions had only a secondary effect. The annealed material showed anisotropy in macroscopic crack growth rate, where the fastest crack growth was observed for the 0°-specimen, in which the crack ran parallel to the laminated structure, and the 60°-specimen showed the slowest crack growth. The anisotropy in crack growth rate almost vanished after spheroidizing heat treatment. Slot et al. [32] studied the influence of the microstructural orientation of low carbon steels on the tensile properties, Charpy impact energy, and fatigue crack growth rate in longitudinal, transversal, and short directions, finding that the microstructure orientation has a statistically significant influence on these properties. The anisotropy in material structure had a small or negligible influence on the yield stress and tensile strength; however, the T-L and T-S anisotropy ratios were approximately 0.4 for the Charpy test, and approximately 1.19 and 0.43 for the crack growth, respectively. Similar results have been observed in other metal alloy systems, such as FSW Al–Li [33], where the tensile strength was higher in the longitudinal direction, as compared to the transverse direction.

While there is a large number of published studies on the anisotropic behavior of steel [34], there is little published research that examines the high-cycle fatigue anisotropy of the API 5L pipeline steel. Because of this, the aim of this study is to obtain and analyze experimental S–N fatigue curves of API 5L X42 pipeline steel in different directions, in order to determine its level of anisotropy, and discuss its relationship with the orientation of the pearlite bands. This knowledge will be useful to perform more accurate mechanical integrity assessments of pipelines operating under directional cyclic loading conditions. The S–N curves were obtained by force-controlled uniaxial fatigue tests with smooth bar specimens in three orientations: longitudinal (L-0°), circumferential (C-90°), and diagonal (D-45°) of an API 5L pipe manufactured by the UOE process, where longitudinal orientation corresponds to the rolling direction.

## 2. Materials and Methods

### 2.1. Material

The material used in the experimentation was extracted from a seam-welded pipe of 24 in. nominal diameter and 0.500 in. nominal thickness of API 5L X42 PSL1 specification manufactured by the UOE process, with normalized heat treatment condition. This pipe was removed from service, with an operational history of 8 years. Table 1 reports the operation conditions of pressure, service, and temperature. The chemical composition of the used material, determined by atomic absorption spectrophotometry, is shown in Table 2.

**Figure 2:**
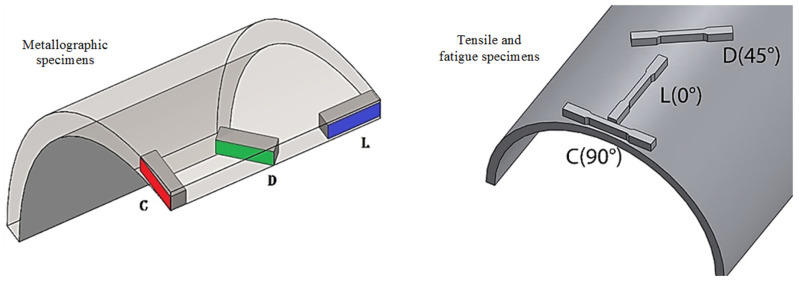
Directions of the test specimens.

Used specimens were extracted from the base material, far from welding beads. Metallographic and fatigue properties were assessed amongst three directions of the pipe: longitudinal (L-0°), parallel to the rolling direction (RD); diagonal (D-45°), inclined 45° with respect to the RD; and circumferential direction (C-90°), perpendicular to the RD. The three orientations are schematically shown in Figure 2.

### 2.2. Microstructural Examination

Microstructural examination was realized for both optical microscopy (Olympus GX-40, Olympus, Tokyo, Japan) and Scanning Electron Microscopy (JEOL JSM6300, JEOL, Tokyo, Japan). Metallographic specimens were etched with Nital 2%. The metallographic preparation was carried out as established in the ASTM E3-11 (2017) [35] and ASTM E407-07 (2015) [36] standards. Specimens oriented in the L-0°, D-45°, and C-90° directions are shown in Figure 1. The pearlite banding degree (*A_i_*) and ferritic grain orientation parameter (*Ω*_12_) were determined according to ASTM E1268-01 (2016) [37] standard. The average grain size was determined according to ASTM E112-13 (2021) [38] standard, and the nonmetallic inclusion content was determined as per ASTM E45-18a [39] standard.

### 2.3. Mechanical Tests

**Figure 3:**
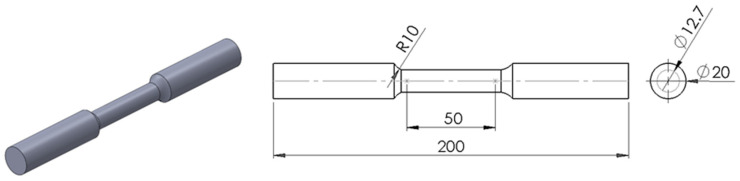
Geometric dimensions of tensile specimens [mm].

Uniaxial tensile tests were carried out according to the ASTM E-8 [40] standard. The test specimens had a gage section of 12.7 mm (½ in) diameter and 50.8 mm (2 in) length as illustrated in Figure 3. Three tests were conducted for each orientation. The tests were conducted at room temperature and humidity (25 °C, 52.8% humidity), at a strain rate of 0.02 min^−1^. The tests were performed in a universal electromechanical testing machine with a 100 kN load cell, an axial extensometer Epsilon model 3542-050M-50-ST, and a data acquisition system.

Hardness Rockwell B [HRBW] tests were performed according to ASTM E18 [41] Std. Tests were performed at ambient temperature, using a Wilson hardness tester (Buehler, Lake Bluff, IL, USA), model 3dttb, and a tungsten carbide ball indenter with a specified diameter of 1/16 in. The preliminary test force was 10 kgf, and the total test force of 100 kgf. Dust, dirt, or other foreign materials were removed from the sample surface by grinding and cleaning according to ASTM E3-11 (2017) [35] sample preparation procedure. At least 10 indentations were performed for each analyzed direction.

### 2.4. Force-Controlled Fatigue Tests

**Figure 4:**
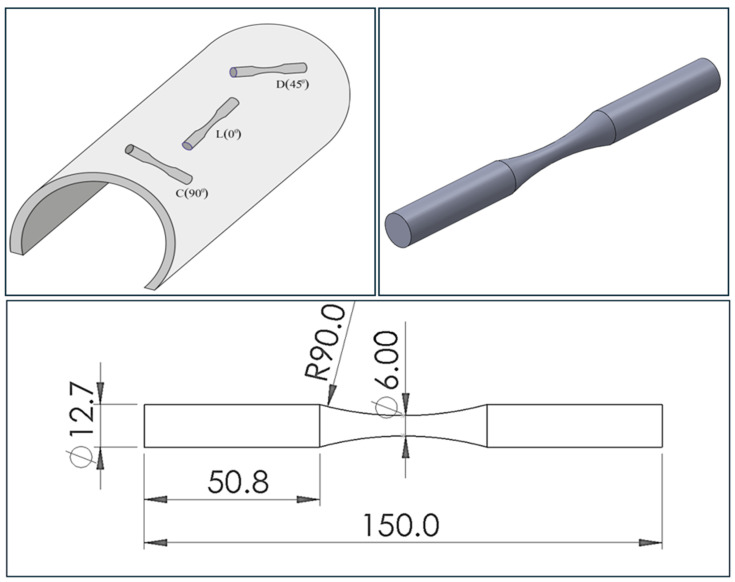
Geometry of the continuous radius specimen for fatigue tests, machined according to the ASTM E-466 standard [mm].

Force-controlled constant amplitude axial fatigue tests were performed according to the ASTM E466 Std. [42], with specimens oriented in the longitudinal (L-0°), diagonal (D-45°), and circumferential (C-90°) directions. The fatigue test specimens were smooth bars, whose geometry and dimensions are shown in Figure 4. The reduced section of the test specimen was ground and polished to achieve a smooth and shiny surface finish as specified in the ASTM E466 Std. To assure the correct surface finish, after polishing, each test specimen was examined under a stereomicroscope at 20×, looking for deep scratches, turning marks, cracks, pores, or corrosion pits.

The axial force-controlled fatigue tests were conducted in a servo-hydraulic test machine equipped with a 100 kN load cell, displacement measurements system (LVDT), and data acquisition system. The force ratio was R = −1, using a sinusoidal waveform load spectrum, at 15 Hz frequency. This experimental arrangement was focused on the life up to crack initiation; therefore, fatigue tests were conducted until a complete fracture occurred, and a visible crack was fully identified at 20× magnification or after 10^6^ cycles were completed. Tests were conducted at room temperature and humidity conditions. The stress amplitude levels were established at 75%, 65%, 55%, and 45% of the average ultimate tensile strength determined in the tensile tests for each specimen orientation. To provide the desired accuracy of statistical inference each stress level was tested in triplicate, using 12 specimens for each evaluated direction and a total of 36 specimens for completing the three S–N curves.

The S–N fatigue data were analyzed by the linearized Stress–Life relationship provided in the ASTM E-739 Std. [43], which has the form:

(1) $$log\left(S\right)\;=\;A\;+\;B\left(logN\right)$$

where *N* is the fatigue life in cycles, *S* is the maximum value of constant-amplitude cyclic stress, *A* is the strength coefficient and *B* is the fatigue strength exponent. The values of these last two parameters were calculated by the linear regression method from the experimental S–N curves plotted in logarithmic scale.

## 3. Results and Discussion

### 3.1. Metallographic Examination

**Figure 5:**
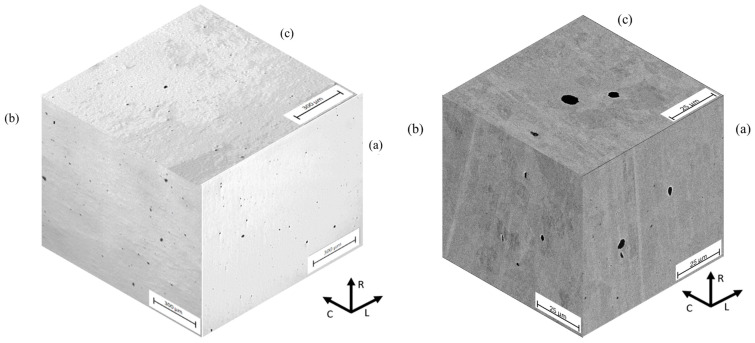
Non-metallic inclusions of API 5L X42 steel: (a) longitudinal (L-90°); (b) circumferential (C-90°); and (c) radial (R) directions. Optical microscopy, unetched.

**Figure 6:**
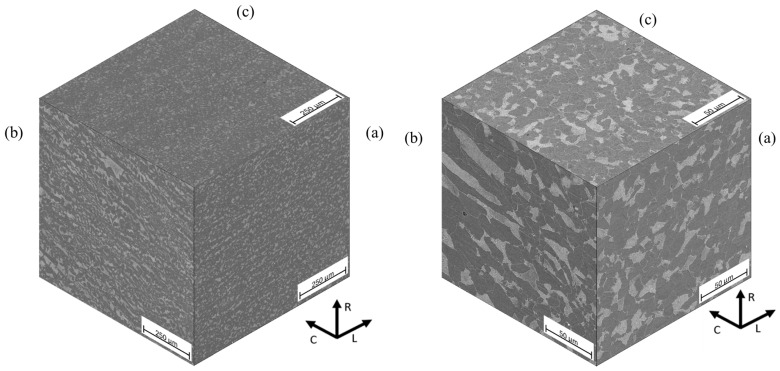
Microstructure of API 5L X42 steel: (a) longitudinal (L-0°); (b) circumferential (C-90°); and (c) radial (R) directions. Scanning electron microscope, SEI bright field, etched with Nital 3.

**Figure 7:**
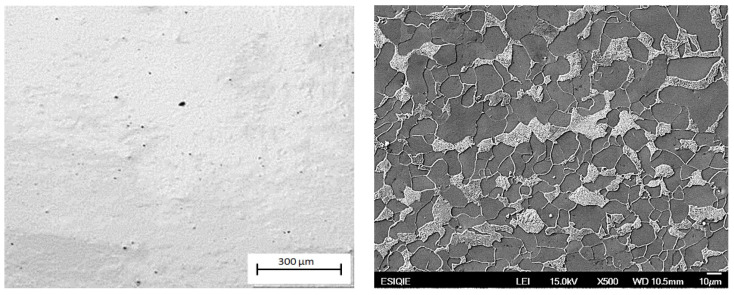
Microstructure of API 5L X42 steel in the D-45° direction.

Figure 5 shows the typical aspect of the nonmetallic inclusions for the longitudinal (L-0°), circumferential (C-90°), and radial (R) directions of the original pipe. The nonmetallic inclusions were identified as globular oxides of type D, with a size range between 10 and 24 μm in the three orientations. Figure 6 shows the typical microstructure in the three pipe’s directions, and Figure 7 shows the microstructure in the D-45° direction. The microstructure is a matrix of ferrite grains of 11 µm average diameter, with lamellar pearlite colonies. Slightly elongated grains and moderate pearlite banding are observed in the L-0° direction (corresponding to the RD). In contrast, the D-45° and C-90° directions showed a nearly homogeneous microstructure with equiaxial grains.

Table 3 shows the stereological parameters of the microstructure, where it is observed that the %Vol. of inclusions, the Ferrite-Pearlite contents, as well as the grain size, are similar in the three directions. The A_i_ and *Ω*_12_ parameters that indicate the degree of pearlite banding are maximum in the L-0° direction, and smaller in the C-90° direction, with intermediate values in the D-45° direction, but closer to the C-90° direction. The observed microstructural differences are attributed to the manufacturing process that produces a banded microstructure in the rolling direction (L-0°), while the normalization heat treatment has a homogenizing effect.

### 3.2. Mechanical Properties

**Figure 8:**
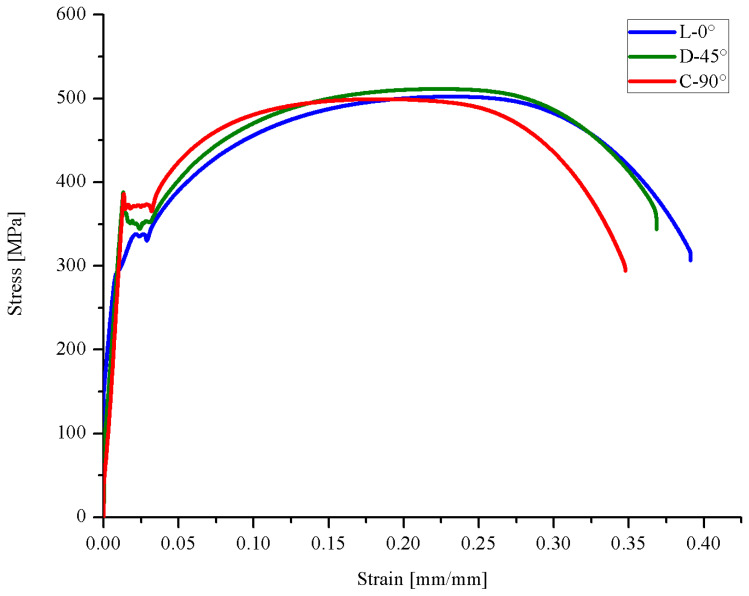
Stress vs. Strain curves for the L-0°, D-45° and C-90° evaluated directions of the API 5L X42 steel.

Figure 8 shows the Stress vs. Strain curves obtained for the three evaluated directions; while Table 4 shows the experimental tensile test data and required tensile strength values for API 5L X42 pipeline steel specification.

For comparison purposes, several tensile strength and hardness properties reported in the literature were collected and presented in Table 5, along with experimental data obtained in this study. It can be observed that hardness tests are not sensitive to variation amongst directions. In contrast, tensile tests show an anisotropic behavior, being the circumferential direction being the strongest orientation in almost all cases, with differences between 5% and 26%.

**Figure 9:**
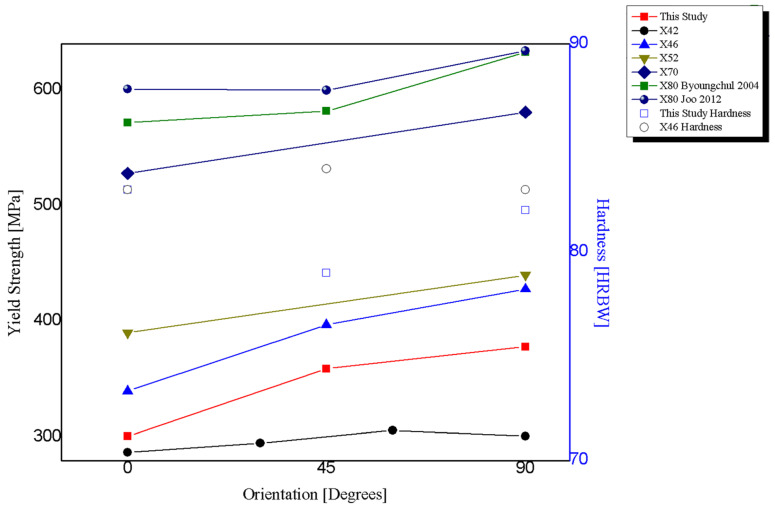
Tensile and hardness properties of pipeline steels [13,23].

**Figure 10:**
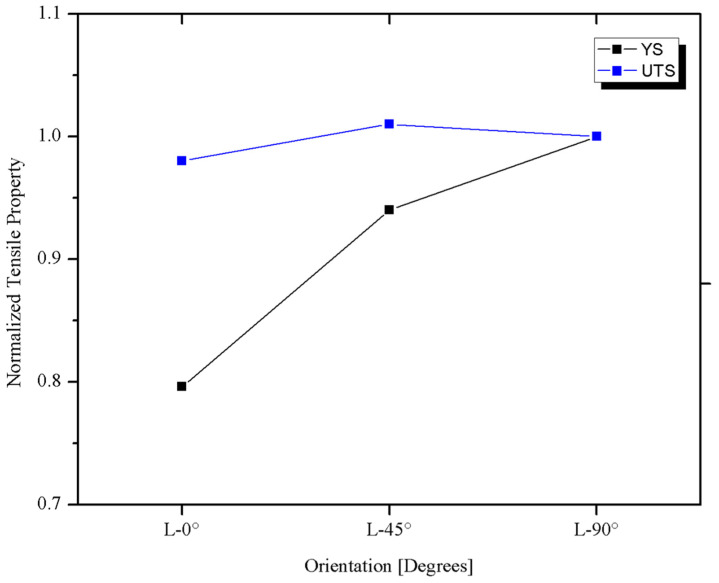
Tensile properties as a function of the test direction. Normalized by the C-90° value.

Tensile results show that the highest tensile and yield strengths are in the C-90° direction, while the L-0° and D-45° directions showed lower values with respect to the C-90°, but very close between them Figure 9 shows the tensile properties of pipeline steels. Figure 10 shows the tensile properties as a function of the test direction, normalized by the C-90° value. The reason for using the C-90° direction as reference is that in internally pressurized pipes, this is the principal stress direction under normal loading conditions, and therefore it is the most important from the design and structural integrity point of view.

As seen in Figure 10, the greatest variation as a function of the test direction was observed in the Yield Strength (*YS*), which is consistent with the results obtained by other researchers [23,25], while the greatest anisotropy of the tensile properties is observed between the C-90° and the D-45° directions, which differs from the reported by others [13,23,44], where the most anisotropic direction was the L-0°. However, the difference between the results of this study and those reported by others in Table 5 is less than 4%, which can be considered within the experimental error.

### 3.3. Fatigue Tests Results and Analysis

**Figure 11:**
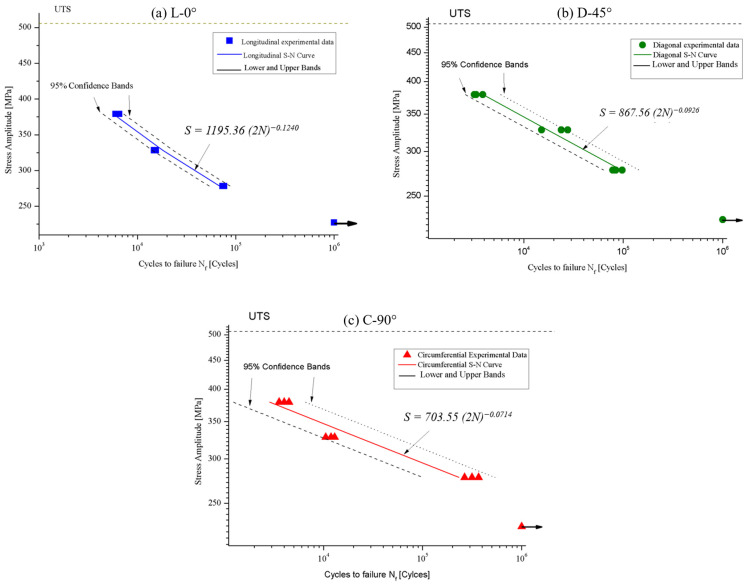
Experimental S–N curves for the API 5L X42 steel, in (**a**) L-0°, (**b**) D-45°, and (**c**) C-90° directions.

**Figure 12:**
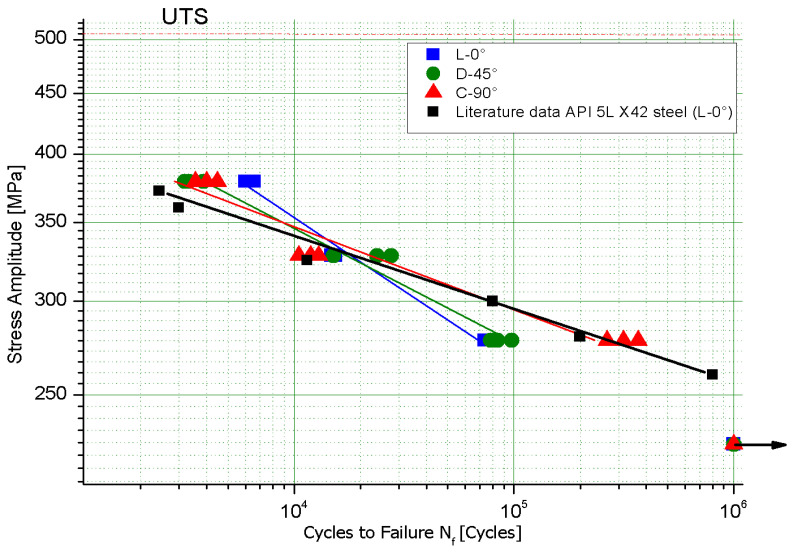
S–N curves for the three test directions of the API 5L X42 steel.

The axial force-controlled fatigue test results of the API 5L X42 steel in the form of S–N curves for the three tested directions are shown in Figure 11. Likewise, Figure 12 shows the S–N curves overlapped, for the three assessed directions. In addition, for comparison porpoises, the *B* exponent was estimated from the fatigue data reported by Hong et al. [45]. The direction evaluated in the mentioned study corresponds to L-0°.

As seen in Figure 11 and Figure 12, there is a clear dependency of fatigue endurance on the test direction. To quantitatively analyze the anisotropy level of the experimental data, the linear fatigue life parameters of the S–N equation of the ASTM E-739 Std. (Equation (1)) were calculated, with the results given in Table 6.

Before going further, it is important to recall that the fatigue strength exponent (*B*) represents the slope of the log(*S*) vs. log(*N*) fatigue curve and therefore is a good indicator of fatigue endurance, because a high value of *B* indicates a low fatigue endurance, particularly at low-stress amplitudes, provided that the parameter *A* is within the same order of magnitude. Thus, the subsequent analysis will be based mostly on this parameter.

It was observed that the *B* values obtained here are higher than those obtained for the published data of Hong et al., but near the typical value reported by the Universal Slopes Coffin–Manson Law (−0.08), indicating that the API 5L X42 steel tested here has a lower fatigue strength as compared to Hong et al. A possible explanation of this result could be that, even when the specification is the same, the steel tested by Hong was less anisotropic and had a more homogeneous microstructure. Furthermore, since the material tested here was extracted from a pipe segment withdrawn from service, it is possible that the material had already accumulated some level of fatigue damage that resulted in shorter fatigue lives.

**Figure 13:**
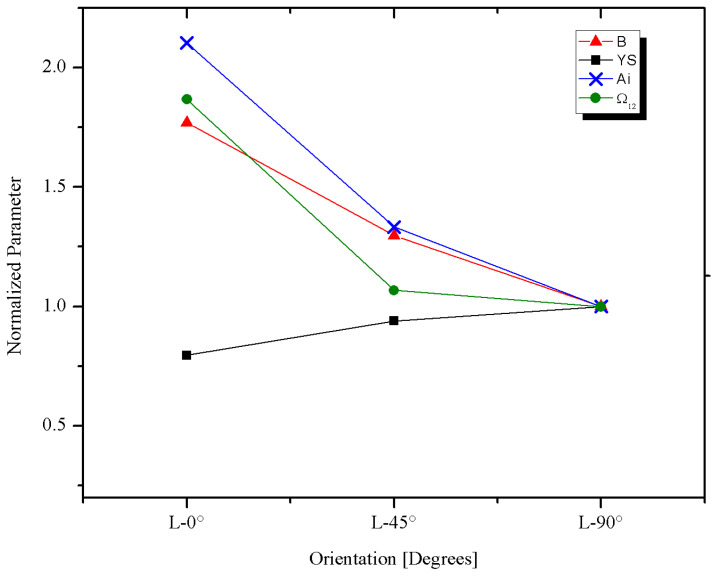
Fatigue strength exponent (*B*), degree of pearlite banding (*A_i_*), ferritic grain orientation parameter (*Ω*_12_), and yield strength (*YS*), normalized by the C-90° as a function of the test direction.

In order to identify the parameter that determines the anisotropy in the API 5L X42 pipeline steel, the linear fatigue life parameters were normalized with respect to the direction C-90°, with the results given in Table 7 and Figure 13.

From Figure 12, it is clear that the parameters that control the anisotropy of the API 5L X42 pipeline steel tested here are the degree of pearlite banding (*A_i_*) and the ferritic grain orientation parameter (*Ω*_12_), because the fatigue parameter *B* follows the same tendency, while the variation of *YS* with respect to the test direction has a much lower effect.

Several authors [12,30,31,32] have suggested that the anisotropic fatigue behavior in low-carbon steel can be attributed to the elongated MnS inclusions that are oriented parallel to the rolling direction. They argue that when the applied principal stress is perpendicular to the rolling direction, the elongated MnS inclusions introduce stress concentration fields that favor fatigue crack nucleation and growth, thus reducing fatigue endurance. However, in the material studied here, the inclusions were of globular oxides, so even though they had different contents in the three directions, the variations did not show any correlation with the fatigue parameter *B*, nor the tensile properties. Therefore, the anisotropy controlling factor identified here was the orientation of the microstructure. The investigation of the causes of this behavior goes beyond the scope of this study, but we foresee that they can be related to two phenomena, according to the fatigue stage that the material is going through. As it is well known, the process of fatigue in metals occurs in two stages, the first one is the damage accumulation, which is controlled by the dislocation arrays that are generated by the cyclic loading, and clearly, these arrays are influenced by the microstructure orientation. The second stage comprises the nucleation and growth of a crack, where the propagation mechanism takes place at a small process zone located ahead of the crack tip, and where the interactions with the microstructure and its orientation are very complex [46].

In this study, the strongest direction, from the fatigue endurance point of view, was the circumferential C-90° direction; it had the lowest value of *B*, the highest tensile properties, and the lowest degree of banding (*A_i_*) and the ferritic grain orientation parameter (*Ω*_12_). Therefore, it can be concluded, so far, that the orientation of the microstructure not only determines the level of anisotropy but also the fatigue endurance, with the tensile properties having a minor influence.

## 4. Summary and Conclusions

The anisotropic behavior of fatigue endurance of API 5L X42 pipeline steel was evaluated by axial force-controlled fatigue tests in smooth bar specimens in three directions: Longitudinal (L-0°), Diagonal (D-45°), and Circumferential (C-90°). Results showed that both the tensile and fatigue endurance behavior are anisotropic, with anisotropy ratios of 1.297 for the D-45° direction and 1.736 for the L-0° direction. In all cases, L-0° direction showed the lowest fatigue life, while the highest was observed in C-90° direction. Fatigue strength exponents exhibited significant directional dependence, which can represent as much as 20% of fatigue lifetime reduction depending on the evaluated direction. These variations establish and modify quantitatively the response relative to crack initiation and consequently on total fatigue life. Therefore, the relative orientation of microstructural features with respect to the applied loads must be considered to estimate the remaining life of structural components.

A linear S–N relationship was used to analyze the results, indicating that the parameters that control the anisotropy of the API 5L X42 pipeline steel tested here are the degree of pearlite banding (*A_i_*) and the ferritic grain orientation parameter (*Ω*_12_), while the variation of *YS* with respect to the test direction had a much lower effect. These facts highlight the effects of banding and grain orientation qualitatively, determining their influence on crack initiation. They also open new questions associated with events where some materials showed lower fatigue strength than the design specifications.

Contrary to the suggestion of other authors, the anisotropic fatigue behavior of the material studied here was not determined by the orientation of the nonmetallic inclusions, basically because the inclusions were globular oxides, whose variations did not show any correlation with the fatigue parameters, nor the tensile properties. Furthermore, the anisotropy controlling factors identified here were the degree of banding (*A_i_*) and the ferritic grain orientation parameter (*Ω*_12_). Although the role of non-metallic inclusions is not ruled out, and considering the wide variety of chemical compositions, microstructures, forming processes, and heat treatments of steels, the effect of microstructural banding and grain orientation is consistently demonstrated in this work.

The causes of the anisotropic fatigue behavior observed here show that the interactions of the fatigue crack tip with the microstructure during the crack propagation stage have a significant effect on the fatigue endurance. The direction (C-90°) with the lowest values of banding degree (*A_i_*) and grain orientation parameter (*Ω*_12_) showed the strongest fatigue endurance behavior (the lowest *B*). However, further research must be conducted to determine if there is any effect of crystallographic texture on fatigue anisotropy of low-carbon steel.

Understanding and accounting for the anisotropic behavior of fatigue in pipeline steels is significant for reliable design and operation. This involves recognizing that the steel’s fatigue endurance varies depending on the direction of the applied load and the crack orientation. By considering the directional dependence of fatigue, more precise fatigue life prediction models for damage tolerance estimation can be developed, rather than relying on isotropic assumptions. Also, detection of potential crack initiation can be optimized by understanding the directional sensitivity of fatigue, developing more effective inspection techniques. Further research is needed, especially for the interaction with external factors such as environment, temperature, or combined damage mechanisms.

## Figures and Tables

**Table 1:** Raw material operation conditions.

Properties/Operation Condition	API 5L X42 Steel
Product Specification Level	PSL1
Transported product	Light crude oil
Nominal Diameter	24 in (609.6 mm)
Nominal Thickness	0.500 in (12.7 mm)
Design Pressure	80 kg/cm^2^ (1138 psi)
Working Pressure	65.3 kg/cm^2^ (929 psi)
Design Temperature	45.0 °C (113 °F)
Working Temperature	22.1 °C (71.78 °F)
Run time	8 years

**Table 2:** Chemical composition of API 5L X42 steel [% wt].

ID	C	S	Mn	Cu	P	Cr	Si	Mo	Ti
Tested Steel API 5L X42	0.220	0.040	1.240	0.023	0.003	0.085	0.260	0.260	0.002
API 5L Spec	0.260 [max.]	0.030 [max.]	1.300 [max.]	0.035 [max.]	0.030 [max.]	-	-	-	0.040 [max.]

**Table 3:** API 5L X42 stereological counting.

Direction	Non-Metallic Inclusions [%]	Ferrite [%]	Pearlite [%]	*A_i_*	*Ω*_12_	Grain Size	
						[μm]	ASTM
L-0°	0.552 ± 0.058	71.54 ± 2.76	28.46 ± 2.76	1.64	0.28	24.4 ± 1.98	8.0
D-45°	0.506 ± 0.036	70.65 ± 2.25	29.35 ± 2.25	1.04	0.16	24.2 ± 2.20	8.0
C-90°	0.533 ± 0.035	70.14 ± 2.07	29.86 ± 2.07	0.78	0.15	29.76 ± 2.50	7.0
R	0.497 ± 0.062	74.15 ± 2.01	25.85 ± 2.01	-	-	10.87 ± 3.61	10.0

**Table 4:** Tensile strength properties obtained for the three evaluated directions of the API 5L X42 steel.

ID	Direction	Diameter	Area	*YS* Load	*YS*	Ultimate Load	*UTS*	Elongation	Ramberg-Osgood Constants	
		[mm]	[mm^2^]	[kN]	[MPa]	[kN]	[MPa]	[%]	Strength Coefficient *K* [MPa]	Strain Hardening Constant *n*
This study	L-0°	12.57	124.10	38.38	309.24	61.06	492.07	40.16	-	-
		12.62	125.09	36.60	292.617	62.85	502.42	38.2	-	-
		12.68	126.28	38.15	302.15	62.74	496.87	39.6	-	-
	Average	12.62 ± 0.06	125.15 ± 1.09	37.71 ± 0.96	301 ± 6.81	62.22 ± 1.01	497 ± 4.26	39.3 ± 0.8	515	0.095
	D-45°	12.68	126.28	45.47	360.1	64.53	510.98	35.92	-	-
		12.66	125.88	46.03	365.69	64.41	511.68	37.65	-	-
		12.59	124.49	43.88	352.44	63.86	512.93	38.18	-	-
	Average	12.64 ± 0.04	123.3 ± 0.94	45.13 ± 1.12	359 ± 6.35	65.26 ± 0.33	512 ± 0.81	37.2 ± 0.96	524	0.079
	C-90°	12.56	123.90	46.22	373.08	61.88	499.42	33.99	-	-
		12.49	122.52	46.82	382.14	62.53	510.39	34.5	-	-
		12.54	123.51	46.84	379.26	62.32	504.56	35.53	-	-
	Average	12.53 ± 0.04	123.31 ± 0.71	46.62 ± 0.35	378 ± 3.77	62.24 ± 0.33	505 ± 4.48	34.7 ± 0.6	516	0.066
Spec.	API 5L X42	-		-	Min. 290	-	Min. 414	Min. 22	-	-

**Table 5:** Tensile and hardness properties of API 5L X42 steel.

ID	Direction	*YS* [MPa]	*UTS* [MPa]	Elongation [%]	Hardness [HRBW]
This study	L-0°	301 ± 8.13	497 ± 5.17	39.2 ± 0.8	83 ± 2
	D-45°	359 ± 6.35	512 ± 0.63	37.2 ± 0.8	79 ± 1
	C-90°	378 ± 4.53	505 ± 5.49	34.7 ± 0.6	82 ± 1
X42 [12]	L-0°	287	-	35.9	-
	D-30°	295	-	35.1	-
	D-60°	306	-	29.5	-
	C-90°	301	-	15.5	-
X46 [25]	L-0°	340 ± 5	505 ± 9	35.4 ± 0.8	83
	D-45°	397 ± 4	517 ± 8	34.9 ± 0.5	84
	C-90°	428 ± 10	532 ± 12	31.7 ± 0.7	83
X52 [44]	L-0°	390	473	31	-
	C-90°	440	536	34	-
X70 [23]	L-0°	528 ± 17	631 ± 5	-	-
	C-90°	581 ± 2	657 ± 5	-	-
X80 [23]	L-0°	572 ± 9	691 ± 3	17.1 ± 1.2	-
	D-45°	582 ± 13	667 ± 6	19.7 ± 0.5	-
	C-90°	633 ± 8	722 ± 4	15.8 ± 1.5	-
X80 [13]	L-0°	601 ± 35	676 ± 4	13 ± 2	-
	D-45°	600 ± 35	663 ± 10	16 ± 2	-
	C-90°	634 ± 29	707 ± 16	15 ± 1	-
Spec.	API 5L X42	Min. 290	Min. 414	Min. 22	-

**Table 6:** Linearized fatigue life parameters of the API 5L X42 steel in the tested directions.

Direction	*B*	*A* [MPa]
C-90°	−0.0714	704
D-45°	−0.0926	868
L-0°	−0.1240	1195
Hong et al. [45]	−0.0606	593

**Table 7:** Normalized properties of the API 5L X42.

Linear Fatigue Life Parameters at 95% Confidence	Specimen Direction		
	L-0°	D-45°	C-90°
*B*(θ)/*B*(C-90°)	1.736	1.297	1.0
*YS*(θ)/*YS*(C-90°)	0.796	0.940	1.0
*A_i_*(θ)/*A_i_*(C-90°)	2.103	1.333	1.0
*Ω*_12_(θ)/*Ω*_12_(C-90)	1.867	1.067	1.0

## Data Availability

The original contributions presented in this study are included in the article. Further inquiries can be directed to the corresponding author.

## Abbreviations, Symbols, and Definitions

The following abbreviations and symbols were used in this manuscript:

*A*	Strength fatigue coefficient
*A_i_*	Banding degree
API	American Petroleum Institute
ASTM	American Society for Testing and Materials
*B*	Fatigue strength exponent
FSW	Friction Stir Welding
HRBW	Hardness Rockwell B
ID	Identification
*K*	Ramberg-Osgood Strength Coefficient
*n*	Ramberg-Osgood Strain Hardening Constant
*N_f_*	Number of cycles to failure
R	Stress ratio
RD	Rolling direction
*S*	Maximum value of constant amplitude cyclic stress
UOE	U-ing, O-ing, and Expanding process
*UTS*	Ultimate Tensile Strength
*YS*	Yield Strength
*Ω*_12_	Microstructural orientation parameter
